# Synergistic effects of PGPRs and fertilizer amendments on improving the yield and productivity of Canola (*Brassica napus* L.)

**DOI:** 10.1186/s12870-025-06062-3

**Published:** 2025-01-14

**Authors:** Haji Muhammad, Muhammad Ijaz, Abdul Sattar, Sami Ul-Allah, Ahmad Sher, Muhammad Asif, Muhammad Dilshad, Khalid Mahmood, Muhammad Waheed Riaz, Muhammad Saqlain Zaheer, Muhammad Rizwan, Salim Manoharadas

**Affiliations:** 1 Asian Institute of Technology, Bangkok, Thailand; 2 Institute of Agronomy, Bahauddin Zakariya University, Multan, Pakistan; 3 Institute of Plant Breeding & Genetics, Bahauddin Zakariya University, Multan, Pakistan; 4 Institute of Agronomy, University of Agriculture, Faisalabad, Pakistan; 5 SAWiE Ecosystems (Pvt Ltd), Punjab, Pakistan; 6 State Key Laboratory of Wheat Breeding, Group of Wheat Quality and Molecular Breeding, College of Agronomy, Shandong Agricultural University, Tai’an, Shandong 271000 China; 7 Department of Agricultural Engineering, Khwaja Fareed University of Engineering and Information Technology, Rahim Yar Khan, Pakistan; 8 Institute of Crop Science and Resource Conservation (INRES), University of Bonn, 53115 Bonn, Germany; 9 Department of Botany and Microbiology, College of Science, King Saud University, Riyadh, 11451 Saudi Arabia

**Keywords:** PGPRs, *Bacillus subtilis*, *Azotobacter salinestris*, Biochar, Compost, Poultry manure, Animal manure, Fertilizers, Improving yield, Canola

## Abstract

**Background:**

Organic fertilizers are safer and more eco-friendly than chemical fertilizers; hence, organic fertilizers can be used to support sustainable farming. The effects of PGPRs are manifold in agriculture, especially in monoculture crops, where the soil needs to be modified to increase germination, yield, and disease resistance. The objective of this study was to assess the effects of PGPRs combined with fertilizer on the yield and productivity of canola. Canola was chosen for its global importance as an oilseed crop and its responsiveness to soil amendments, making it ideal for evaluating the synergistic effects of PGPRs and fertilizers on yield and soil health.

**Methodology:**

This research, which was carried out over two years, was aimed at establishing the effectiveness of PGPRs together with organic and inorganic fertilizers on canola yields and was performed with a two-factorial RCBD design under field conditions. We applied *Azotobacter salinestris* and *Bacillus subtilis* with biochar, compost, animal manure, poultry manure, and NPK fertilizer. Insect pest management and other agronomic practices were carried out to maintain the experiment.

**Results:**

Canola yield and agronomic traits were enhanced by the combination of *Bacillus subtilis* with the fully recommended N: P:K ratio (140:55:40 kg/ha). Additionally, the application of *Bacillus subtilis* with biochar at 2 tons/ha improved the yield and quality of canola, as well as the structure and nutrient regulation of the soil.

**Conclusion:**

In light of these results, we recommend the application of *Bacillus subtilis* to canola seeds along with either 2 t/ha biochar or the entire recommended dose of N: P:K (140:55:40 kg/ha). These strategies are sustainable and help producers and the environment increase the productivity of canola. Combining PGPRs with fertilizers for canola enhances nutrient efficiency, promotes sustainable growth, and boosts stress resilience, addressing agricultural and environmental challenges.

**Clinical trial number:**

Not Applicable.

## Introduction

Oil crops are rich sources of protein, vitamins, dietary fibers, minerals, cooking oil, and other raw materials [1]. Canola (*Brassica napus* L.) is a cultivated oil crop species of the Brassicaceae family worldwide [2]. China is among the 3rd largest canola producers in the world, with a yield of 13.1 million metric tons from 2019 to 2020 [3]. Canola is still ranked after soybean in terms of oil production [4]. In Pakistan, canola is cultivated in all provinces over 26.02 thousand hectares, with an annual production of 102 thousand tonnes from 2018 to 19 [5]. Canola plants, ranging from 100 to 150 cm in height, with some varieties reaching 180 cm, have alternate, simple, and lanceolate leaves with a waxy coating and a length of 20–40 cm and exhibit a typical Brassicaceae inflorescence with bright yellow flowers arranged in a race, allowing both self-pollination and cross-pollination [6]. The plants produce slender pods that are 5–10 cm long, contain 10–20 spherical seeds that are 1.5–2.5 mm in diameter and brown or black, and have an erect main stem with 2–4 branching levels and a diameter of 1–2 cm [7]. Hence, the taproot system together with lateral roots and extending to a depth of 60–120 cm bears the whole plant in the soil [8].

Plant growth promoting rhizobacteria (PGPR) are bacteria that inhabit the rhizosphere of plants; their beneficial effects include improved nutrient acquisition and root modification through plant hormone control. The use of PGPR in agriculture enhances crop yields and reduces pollution, hence enhancing ecological and economic security [9]. PGPRs are essential to sustainable agriculture since they contribute to providing a healthier and more productive food system [10]. Through the process of biomass pyrolysis, biochar has several uses, with the principal uses being as follows: This material has several uses, with the most important one being as follows: In agriculture, biochar has been largely used to increase soil fertility, promote plant germination, and provide crops with nutrients. They asserted that the documented outcomes marked the intervention as efficient; therefore, it yielded positive results [11]. Thus, it enhances farming productivity and, consequently, increases the results of agricultural activities [12]. Compost is a product of the natural process of decomposing natural organic material, which is characterized by humus and valuable nutrients that ameliorate all aspects of the physical, chemical, and biological properties of soils [13]. Compost enhances the ability of the soil to hold other nutrients since it increases the CEC of the soil and supplies fundamental nutrients such as nitrogen, phosphorus, and potassium to the plants [14]. When compost is incorporated into the soil, nutrient holding and nutrient delivery capacity are increased, resulting in improved plant health [15].

The residues of animal faces, urine together with plant material, and manure are important for farming because they provide nutrients for plants and increase the quality of the organic matter and tillage of the soil [16]. The use of immature and composted manure on croplands improves the conditions of the upper soil layer and its ability to hold water and nutrients, increases microbial activity, and supplies vital minerals and plant nutrients [9]. By using manure as a natural fertilizer, farmers can help soil and crops grow healthily and naturally [17]. It is a fulfilling source of nutrients, especially nitrogen, and hence quickly becomes a favorite fertilizer for nitrogen-deficient soils and is even richer in nitrogen, phosphorous, and potassium than all other animal manures [18]. The products produced from pelletized chicken manure may contain additional nutrients; therefore, it is regarded as an effective fertilizer for encouraging the production of green leaves in plants [19]. Animal droppings, such as poultry manure, are rich in nitrogen, phosphorus, potassium, and other nutrients that help in both the growth and development of plant crops [20].

The problem of low-productivity canola in Pakistan is, therefore, widely embodied by several factors [21]. Global warming and an increase in weather variability affect canola outputs, and unpredicted weather events lead to poor germination; low soil health and poor topsoil formation through erosion and/or acidification erode soil fertility and reduce the water holding capacity [22]. Poor water supply and water use have compounded this problem, resulting in a scarcity of water and poor plant growth [23]. The seeds that are used are often of low quality and are mostly obtained from farmer’s fields, while the seed treatment is often poor, which leads to low germination rates and poor seedling development [24]. Unfavorable growing conditions such as planting at the wrong time, planting at the wrong density, and providing poor weed control for pests and diseases are possible [25]. Furthermore, poor uptake and distribution of fertilizers and low nutrient status in the soil result in poor plant growth and development [26]. To address these factors, this two-year research study aimed to determine the effects of the integrated application of biochar, compost, poultry manure, animal manure, and chemical fertilizer with PGPRs on canola yield and other traits [27]. Thus, considering the cumulative interaction of these treatments, this study revealed the best interactions that are beneficial for increasing canola yield, improving soil health, and promoting the adoption of sustainable agriculture in Pakistan [28]. The key objectives of this study were to evaluate the synergistic effects of *Azotobacter salinestris* and *Bacillus subtilis* combined with various organic and inorganic fertilizers on the yield and productivity of canola and to assess their impact on soil health and nutrient efficiency. The hypothesis was that the integration of PGPRs with fertilizer amendments would significantly enhance canola yield, agronomic traits, and soil quality compared to fertilizers alone.

## Materials and methods

### Experimental site

The area of study included the research area of the College of Agriculture, Bahauddin Zakariya University (BZU), Bahadur Subcampus Layyah; geographically, it is situated at 30° 58′ 49′′ N latitude and 70° 57′ 57′′ E longitude in southern Punjab, Pakistan, at an altitude of 147 m above the mean sea level. The type of soil identified was sandy with 0. A total of 56% organic matter was present, and the pH was slightly basic and equal to 8.2 [29]. They also described the soil of Layyah as sandy loam, which provides moderate drainage capacity and holds a moderate amount of standing water; hence, it can support several crops [30]. The soil pH was 7.1 is slightly below the alkaline level, which influences nutrient solubility and microbiological processes occurring in the context of soil. Hence, the sandy loam soil of Layyah, which was found to be chemically basic in constitution, needs wise inputs to increase its fertility and yield.

### Experimental layout and inputs

A real experimental setting was established during the past two years (2022–2023, 2023–2024) by adopting a randomized complete block design (RCBD) with two factorials with three replications and 21 plots in each replication, resulting in a total of sixty-three plots. Every plot was 3 m × 1 m, the row-to-row distance was 40 cm, the plant-to-plant distance was 10 cm, and HC-022B hybrid canola was used. The seeds of HC-022B hybrid canola were sourced from Punjab Certus Seed Kanzo Combagro Evyol Group, which specializes in high-yield canola hybrids. *Azotobacter salinestris* and *Bacillus subtilis* cultures for PGPR treatments were obtained from Ayub Agricultural Research Institute, Faisalabad. The experimental design employed a 3 × 7 factorial arrangement comprising two factors. The factors that were targeted by the experiment included the level of plant growth-promoting rhizobacteria (PGPR) (Factor A) and the aspect of fertilizer amendments (Factor B). Factor A consisted of three PGPR levels: PGPR0 received 0 PGPR, PGPR1 was inoculated with *Azotobacter salinestris*, and PGPR2 was inoculated with *Bacillus subtilis* Factor B comprised seven fertilizer amendment treatments: the control, no fertilizers or amendments added, fully recommended fertilizers (FFs), half recommended fertilizers (HFs), biochar (BC), compost (CP), poultry manure (PM), and animal manure (AM). The seedbed was prepared with 2 tons of biochar per hectare, 3 tons of compost per hectare, 2 tons of poultry manure per hectare, and 2 tons of animal manure per hectare. Seeds were immersed in PGPRs, *Azotobacter salinestris* and *Bacillus subtilis* B. The crop was planted on 3rd November 2021 at a rate of 0.81 kg/ha, and weed management was performed manually. The crop was treated with the recommended full dose of 140:55:40 kg/ha N: P:K and half of the recommended rate. Pest management was performed by applying multinational brands containing imidacloprid and bifenthrin at 600 ml/ha according to established guidelines and protocols [31]. Thinning was carried out at 20 and 35 days after sowing for proper plant spacing, irrigation was recommended, and other agronomic practices were followed. The crop was allowed to mature and be harvested on 28 April 2022, spread on a clean surface area for sun-drying for 10 consecutive days, and then threshed to estimate the grain yield. The above treatments were repeated in the second year of the experiment via similar methods of agronomic practices, harvesting, and data collection. The specific dosages of biochar (2 t/ha), compost (3 t/ha), poultry manure (2 t/ha), and animal manure (2 t/ha) were selected based on their nutrient profiles and prior agronomic recommendations for sandy loam soils like those at the experimental site. These dosages were optimized to enhance soil fertility, structure, and microbial activity, creating favorable conditions for canola growth and maximizing the benefits of PGPR applications. The seeds were immersed in freshly prepared cultures of *Azotobacter salinestris* and *Bacillus subtilis* at a concentration of 10⁸ CFU/mL for 30 min to ensure thorough coating. This method of inoculation was employed to enhance the adhesion of PGPRs to the seed surface, facilitating early colonization of the rhizosphere post-germination. This approach aims to optimize nutrient uptake, promote root development, and improve overall plant growth through the synergistic effects of the PGPRs.

### Data collection

The following parameters were measured and recorded in this experiment: plant height, number of primary branches, number of secondary branches, pod length, number of pods per plant, number of seeds per pod, biological yield, and grain yield, which provide a clear picture of the growth and yield of the plants. Randomized sampling was used to measure parameters of plant growth and productivity [32]. Agronomic data from each plot were collected via random sampling, with five plants per plot within a 1 m × 1 m area, plant height, measured from the soil surface to the highest point of the ear via a meter rod. The average height was obtained for all the plots. Five plants from each plot were randomly chosen to record and measure primary and secondary branches, pods, and pod length. Thus, the average value was determined for each parameter. To assess the biological yield, whole plants from a 1 m × 1 m quadrat from each plot were weighed, whereas to estimate the grain yield, plants from that same area were cut and threshed, and the grains were weighed [31]. Thus, the average values of both parameters were determined. This sampling design made it easy to classify and obtain a representative sample of the plant growth and productivity parameters [19]. These parameters were employed to measure the impacts of various treatments on plant growth and yield. The experiments were conducted thrice to reduce the effect of errors and increase the reliability of the results.

### Statistical analysis

The quantitatively collected data were subjected to two-way analysis of variance (ANOVA) with a randomized complete block (RCB) design with a factorial arrangement. All the statistical tests were conducted via Statistix 8.1 software. A post hoc test with the least significant difference (LSD) test was used to analyze significant differences in the treatment means at a probability level of 0. 05, which was significant on the basis of the obtained F test value.

## Results

Canola plant height, the number of primary branches, the number of secondary branches, the number of pods per plant, the number of seeds per plant, biological yield, and grain yield were significantly influenced by two-way interactions, PGPRs, and fertilizer amendments in year 1. However, pod length was not affected by the treatment effects due to the two-way interaction effect between PGPRs and fertilizer amendments in the year-1 experiment, as indicated in Table 1. Thus, when the experiment was repeated in year 2, the plant height, number of primary branches, number of secondary branches, number of pods per plant, pod length, biological yield, and grain yield were highly significantly influenced by the two-way interaction between the PGPRs and the fertilizer amendments. However, the number of seeds per pod was significantly influenced by the two-way interaction between PGPRs and fertilizers, as depicted in Table 1.

**Table 1:** Significance levels of two-way ANOVA of the synergistic effects of plant growth-promoting rhizobacteria (PGPRs) and fertilizer amendments on plant height, number of primary branches, number of secondary branches, number of pods per plant, number of seeds per plant, pod length, biological yield and grain yield of canola

Parameters	Plant Growth Promoting Rhizobacteria (PGPR)	Fertilizer amendments	PGPR*Fertilizer amendments
Year-1, Morphological and reproductive parameters			
Plant Height (cm)	**	**	**
primary branches	**	**	**
Secondary branches	**	**	**
Pods per plant	**	**	**
Seeds per pod	**	**	**
Pod length (cm)	**	**	ns
Biological yield (t/ha)	**	**	**
Grain yield (t/ha)	**	**	**
Year-2, Morphological and reproductive parameters			
Plant Height (cm)	**	**	**
Primary branches	**	**	**
Secondary branches	**	**	**
Pods per plant	**	**	**
seeds per pod	**	**	*
Pod length (cm)	**	**	**
Biological yield (t/ha)	**	**	**
Grain yield (t/ha)	**	**	**

*represents significant (*P* ≤ 0.05), ** represents highly significant (*P* ≤ 0.05), and ns represents nonsignificant (*P* ≤ 0.05)

### Effects on plant height

In year 1, the average plant height of canola was observed to be a maximum of 166 cm by the combined application of PGPR2 (*Bacillus subtilis*) with the fully recommended fertilizer, followed by 165 cm due to the application of biochar with PGPR2 (*Bacillus subtilis*). The lowest value of 132 cm was observed in the control treatment, where no PGPR or fertilizer amendments were applied. The plant height was 23.5% and 23.03% greater than that in the control treatment because of the combined application of the fully recommended fertilizer (N: P:K@140:55:60) kg/ha with PGPR2 (*Bacillus subtilis*), followed by the application of biochar with PGPR2 (*Bacillus subtilis*), as shown in Table 2. Similarly, in year 2, the average plant height of canola was observed to be a maximum of 169 cm by the combined application of PGPR2 (*Bacillus subtilis*) with the fully recommended fertilizer, followed by 167 cm due to the application of biochar with PGPR2 (*Bacillus subtilis*). The lowest value of 130 cm was observed in the control treatment, where no PGPR or fertilizer amendments were applied. The plant height was 23.07% and 22.15% greater than that in the control treatment because of the combined application of the fully recommended fertilizer (N: P:K@140:55:60) kg/ha with PGPR2 (*Bacillus subtilis*), followed by the application of biochar with PGPR2 (*Bacillus subtilis*), as shown in Table 3.

**Table 2:** The synergistic effects of plant growth-regulating rhizobacteria (PGPR) and fertilizer amendments on plant height and the number of primary and secondary branches of canola. (1st year of the experiment)

Factors & Treatments		Plant Height (cm)	Number of primary branches	Number of secondary branches
Control	PGPR0	127 ± 0.26q	3 ± 0.13o	3 ± 0.12 l
	PGPR1	130 ± 0.74p	3 ± 0.26o	3.58 ± 0.11kl
	PGPR2	132 ± 1.21o	3.5 ± 0.11n	4 ± 0.09k
Half Fertilizers	PGPR0	132 ± 0.98o	4 ± 0.09 m	5 ± 0.24j
	PGPR1	134 ± 1.12n	4 ± 0.31 m	5.5 ± 0.36j
	PGPR2	136 ± 1.42 l	4.5 ± 0.42k	7 ± 0.19 l
Animal Manure	PGPR0	135 ± 0.56 m	4.2 ± 0.17 l	8 ± 0.38 h
	PGPR1	142 ± 1.23k	4.2 ± 0.28 l	8.5 ± 0.21 h
	PGPR2	142 ± 2.09k	5 ± 0.15j	10 ± 0.66 g
Compost	PGPR0	145 ± 1.54j	5 ± 0.13j	10 ± 0.45 g
	PGPR1	151 ± 1.78i	5.5 ± 0.05i	11 ± 0.17f
	PGPR2	153 ± 0.89 h	6 ± 0.24f	12 ± 0.53e
Poultry Manure	PGPR0	153 ± 1.45 h	5.6 ± 0.29 h	12 ± 0.43e
	PGPR1	154 ± 1.67 g	5.7 ± 0.06 g	13 ± 0.33d
	PGPR2	156 ± 0.56e	7 ± 0.02c	14 ± 0.12c
Biochar	PGPR0	155 ± 0.88f	6.2 ± 0.01e	13 ± 0.11d
	PGPR1	155 ± 1.20f	6.5 ± 0.31d	13.5 ± 0.72 cd
	PGPR2	160 ± 1.34d	7.5 ± 0.5b	15 ± 0.79b
Full Fertilizers	PGPR0	162 ± 0.98c	7 ± 0.23c	14 ± 0.18c
	PGPR1	163 ± 1.25b	7.5 ± 0.16b	14 ± 0.11d
	PGPR2	166 ± 1.45a	8.1 ± 0.18a	17 ± 0.03a

PGPP0 (no PGPR applied), PGPR1 (*Azotobacter salinestris*), and PGPR2 (*Bacillus subtilis*) were used. The 1st year means are statistically similar on the basis of the least significance difference test at *P* **≤** 0.05; ± standard errors

**Table 3:** Synergistic effects of plant growth-regulating rhizobacteria (PGPR) and fertilizer amendments on plant height, number of primary branches, and number of secondary branches of canola (2nd year of the experiment)

Factors		Plant Height (cm)	Number of primary branches	Number of secondary branches
Control	PGPR0	130 ± 2.34q	4 ± 0.07n	4 ± 0.13 l
	PGPR1	133 ± 1.23p	4 ± 0.89n	4.58 ± 0.06kl
	PGPR2	135 ± 1.20o	4.5 ± 0.45 m	5 ± 0.07k
Half Fertilizers	PGPR0	135 ± 2.12o	5 ± 0.67 l	6 ± 0.09j
	PGPR1	137 ± 2.1n	5 ± 0.56 l	6.5 ± 0.12j
	PGPR2	139 ± 1.19 l	5.5 ± 0.25j	8 ± 0.19i
Animal Manure	PGPR0	138 ± 1.34 m	5.25 ± 0.38k	9 ± 0.05 h
	PGPR1	145 ± 1.19k	5.25 ± 0.68k	9.5 ± 0.23 h
	PGPR2	145 ± 0.18k	6 ± 0.08i	11 ± 0.02 g
Compost	PGPR0	148 ± 2.11j	6 ± 0.34i	11 ± 0.18 g
	PGPR1	154 ± 1.15i	6.5 ± 0.22 h	12 ± 0.34f
	PGPR2	156 ± 1.25 h	7 ± 0.07f	13 ± 0.28e
Poultry Manure	PGPR0	156 ± 0.27 h	6.56 ± 0.26 h	13 ± 0.29e
	PGPR1	157 ± 0.54 g	6.76 ± 0.78 g	14 ± 0.25d
	PGPR2	159 ± 0.29e	8 ± 0.13c	15 ± 0.17c
Biochar	PGPR0	158 ± 0.79f	7.2 ± 0.89e	14 ± 0.05d
	PGPR1	158 ± 1.16f	7.5 ± 0.21d	14.5 ± 0.02 cd
	PGPR2	163 ± 1.34d	8.5 ± 0.31b	16 ± 0.34b
Full Fertilizers	PGPR0	165 ± 2.18c	8 ± 0.29c	15 ± 0.42c
	PGPR1	166 ± 0.03b	8.5 ± 0.56b	15 ± 0.23c
	PGPR2	169 ± 1.02a	9.1 ± 0.41a	18 ± 0.08a

PGPP0 (no PGPR applied), PGPR1 (*Azotobacter salinestris*), and PGPR2 (*Bacillus subtilis*) were used. The 2nd year means are statistically similar on the basis of the least significance difference test at *P* **≤** 0.05; ± standard errors

### Effects on primary branches

In year 1, the average number of primary branches of canola was 8.1 when PGPR2 (*Bacillus subtilis*) was combined with the fully recommended fertilizer, followed by 7.5 when biochar combined with PGPR2 (*Bacillus subtilis*) was applied. The lowest value of 3 was observed in the control treatment, where no PGPR or fertilizer amendments were applied. The primary branches were significantly 62.96% and 60% greater than those in the control treatment because of the combined application of the fully recommended fertilizer (N: P:K @140:55:60) kg/ha with PGPR2 (*Bacillus subtilis*), followed by the application of biochar with PGPR2 (*Bacillus subtilis*), as shown in Table 2. Similarly, in year 2, the average number of primary branches of canola was 9.1 when PGPR2 (*Bacillus subtilis*) was combined with the fully recommended fertilizer, followed by 8.53 when biochar combined with PGPR2 (*Bacillus subtilis*) was applied. The lowest value of 4 was observed in the control treatment, where no PGPR or fertilizer amendments were applied. The average number of primary branches was 56.04% and 53.10% greater than that in the control treatment because of the combined application of the fully recommended fertilizer (N: P:K@140:55:60) kg/ha with PGPR2 (*Bacillus subtilis*), followed by the application of biochar with PGPR2 (*Bacillus subtilis*), as shown in Table 3.

### Effects on secondary branches

In year 1, the average number of secondary branches of canola was observed to be a maximum of 17 by the combined application of PGPR2 (*Bacillus subtilis*) with the fully recommended fertilizer, followed by 15 due to the application of biochar with PGPR2 (*Bacillus subtilis*). The lowest value of 3 was observed in the control treatment, where no PGPR or fertilizer amendments were applied. The primary branches were significantly greater (82.35% and 80% higher, respectively) than those in the control treatment due to the combined application of the fully recommended fertilizer (N: P:K @140:55:60) kg/ha with PGPR2 (*Bacillus subtilis*), followed by the application of biochar with PGPR2 (*Bacillus subtilis*), as shown in Table 2. Similarly, in year 2, the average number of secondary branches of canola was a maximum of 18 following the combined application of PGPR2 (*Bacillus subtilis*) with the fully recommended fertilizer, followed by 16 following the application of biochar with PGPR2 (*Bacillus subtilis*). The lowest value of 4 was observed in the control treatment, where no PGPR or fertilizer amendments were applied. The average number of secondary branches was 77.77% and 75% greater than that in the control treatment because of the combined application of the fully recommended fertilizer (N: P:K@140:55:60) kg/ha with PGPR2 (*Bacillus subtilis*), followed by the application of biochar with PGPR2 (*Bacillus subtilis*), as shown in Table 3.

### Effects on the number of pods

In year 1, the average number of pods per plant of canola was observed to be a maximum of 304 by the combined application of PGPR2 (*Bacillus subtilis* L.) with the fully recommended fertilizer, followed by 285 due to the application of biochar with PGPR2 (*Bacillus subtilis*). The lowest 135 was observed in the control treatment, where no PGPR or fertilizer amendments were applied. The number of pods was significantly greater (55.59% and 52.63%) than that in the control treatment because of the combined application of the fully recommended fertilizer (N: P:K@140:55:60) kg/ha with PGPR2 (*Bacillus subtilis*), followed by the application of biochar with PGPR2 (*Bacillus subtilis*), as shown in Table 4. Similarly, in year 2, the average number of pods per plant of canola was observed to be a maximum of 310 by the combined application of PGPR2 (*Bacillus subtilis*) with the fully recommended fertilizer, followed by 290 due to the application of biochar with PGPR2 (*Bacillus subtilis*). The lowest value of 140 was observed in the control treatment, where no PGPR or fertilizer amendments were applied. The number of pods was 54.83% and 51.72% greater than that in the control treatment because of the combined application of the fully recommended fertilizer (N: P:K@140:55:60) kg/ha with PGPR2 (*Bacillus subtilis*), followed by the application of biochar with PGPR2 (*Bacillus subtilis*), as shown in Table 5.

**Table 4:** Synergistic effects of plant growth-regulating rhizobacteria (PGPR) and fertilizer amendments on the number of pods per plant, number of seeds per pod, and pod length (cm) of canola. (1st year of the experiment)

Factors & Treatments		Number of pod per plant	Number of seed per pod	Pod length (cm)
Control	PGPR0	135 ± 1.96r	6 ± 0.13j	3.66 ± 0.09n
	PGPR1	135 ± 2.56r	9 ± 0.56i	4 ± 0.16mn
	PGPR2	137 ± 1.02r	10 ± 0.03hi	4.41 ± 0.07 l-m
Half Fertilizers	PGPR0	145 ± 0.98q	9 ± 0.21i	4.41 ± 0.41 l-m
	PGPR1	200 ± 1.67k	10.33 ± 0.22 g-i	4.91 ± 0.02k-m
	PGPR2	225 ± 1.78f	10.66 ± 0.32 g-i	5.33 ± 0.04j-l
Animal Manure	PGPR0	155 ± 0.09p	12 ± 0.01 fgh	5.5 ± 0.11i-k
	PGPR1	205 ± 0.87j	12.33 ± 0.28e-g	5.83 ± 0.01 h-k
	PGPR2	235 ± 1.34e	13.33 ± 0.31d-f	6.16 ± 0.06 g-j
Compost	PGPR0	165 ± 0.27o	13.33 ± 0.12d-f	6.16 ± 0.08 g-j
	PGPR1	210 ± 1.14i	14 ± 0.31c-f	6.33 ± 0.02f-i
	PGPR2	250 ± 0.45d	14 ± 0.06c-f	6.5 ± 0.03f-h
Poultry Manure	PGPR0	175 ± 0.35n	14 ± 0.29c-f	6.41 ± 0.02f-i
	PGPR1	215 ± 1.78 h	14.33 ± 0.34c-e	7.5 ± 0.08c-e
	PGPR2	270 ± 0.09c	14.33 ± 0.02c-e	6.91 ± 0.05e-g
Biochar	PGPR0	185 ± 2.11 m	15 ± 0.96b-c	7.25 ± 0.03d-f
	PGPR1	220 ± 1.92 g	15.33 ± 0.67a-d	7.51 ± 0.09c-e
	PGPR2	285 ± 2.33b	15.66 ± 0.83a-c	8.08 ± 0.13b-c
Full Fertilizers	PGPR0	195 ± 1.33 l	16 ± 0.45a-c	8.33 ± 0.19a-c
	PGPR1	225 ± 0.98f	16.66 ± 0.13ab	8.5 ± 0.02ab
	PGPR2	304 ± 0.23a	17.33 ± 0.08a	9.16 ± 0.18a

PGPP0 (no PGPR applied), PGPR1 (*Azotobacter salinestris*), and PGPR2 (*Bacillus subtilis*) were used. The 1st year means are statistically similar on the basis of the least significance difference test at *P* **≤** 0.05; ± standard errors

**Table 5:** Synergistic effects of plant growth-regulating rhizobacteria (PGPR) and fertilizer amendments on the number of pods per plant, number of seeds per pod, and pod length (cm) of canola. (2nd year of the experiment)

Factors		Number of pod per plant	Number of seed per pod	Pod length (cm)
Control	PGPR0	140 ± 2.11 m	7 ± 0.01j	4 ± 0.46k
	PGPR1	140 ± 1.23 m	10 ± 0.19i	4.25 ± 0.36jk
	PGPR2	17.33 ± 2.97i-m	11 ± 0.34hi	4.5 ± 0.27j
Half Fertilizers	PGPR0	150 ± 1.67 lm	10 ± 0.54i	4.5 ± 0.08j
	PGPR1	205 ± 1.98e-j	11.33 ± 0.18 g-i	5 ± 0.34i
	PGPR2	230 ± 2.11d-f	11.67 ± 0.02 g-i	6 ± 0.05b
Animal Manure	PGPR0	160 ± 0.19k-m	13 ± 1.34f-h	5.5 ± 0.02 h
	PGPR1	210 ± 2.44e-i	13.33 ± 0.28e-g	6 ± 0.35 g
	PGPR2	240 ± 1.47c-e	14.33 ± 0.88d-f	6.25 ± 0.78e
Compost	PGPR0	170 ± 1.89j-m	14.33 ± 0.45d-f	6.5 ± 0.32f
	PGPR1	215 ± 1.45e-h	15 ± 0.39c-f	6.5 ± 0.54f
	PGPR2	255 ± 1.78b-d	15 ± 0.36c-f	6.5 ± 0.33f
Poultry Manure	PGPR0	180 ± 2.09 h-l	15 ± 69c-f	7 ± 0.65e
	PGPR1	220 ± 2.45d-g	15.33 ± 1.11c-e	7.5 ± 0.71d
	PGPR2	275 ± 0.15a-c	15.33 ± 0.32c-e	7 ± 0.23 fg
Biochar	PGPR0	190 ± 1.24 g-k	16 ± 0.78b-d	7.5 ± 0.36d
	PGPR1	225 ± 1.29d-f	16.33 ± 0.38a-d	7.76 ± 0.11d
	PGPR2	290 ± 0.31ab	16.67 ± 1.13a-c	9 ± 0.24 g
Full Fertilizers	PGPR0	200 ± 1.36f-i	17 ± 1.15a-c	8.5 ± 0.04c
	PGPR1	230 ± 1.34d-f	17.67 ± 0.34ab	8.75 ± 0.56bc
	PGPR2	310 ± 2.33a	18.33 ± 1.37a	9.5 ± 0.09a

PGPP0 (no PGPR applied), PGPR1 (*Azotobacter salinestris*), and PGPR2 (*Bacillus subtilis*) were used. The 2nd year means are statistically similar on the basis of the least significance difference test at *P* **≤** 0.05; ± standard errors

### Effects on the number of seeds per pod

In year 1, the average number of seeds per pod of canola was observed to be a maximum of 17.33 when PGPR2 (*Bacillus subtilis*) was combined with the fully recommended fertilizer, followed by 16.66 when biochar combined with PGPR2 (*Bacillus subtilis*) was applied. The lowest value of 6 was observed in the control treatment, where no PGPR or fertilizer amendments were applied. The number of seeds per pod was significantly (65.37% and 63.98%) greater than that in the control treatment because of the combined application of the fully recommended fertilizer (N: P:K@140:55:60) kg/ha with PGPR2 (*Bacillus subtilis*), followed by the application of biochar with PGPR2 (*Bacillus subtilis*), as shown in Table 4. Similarly, in year 2, the average number of seeds per pod of canola was observed to be a maximum of 18.33 when PGPR2 (*Bacillus subtilis*) was combined with the fully recommended fertilizer, followed by 17.67 when biochar combined with PGPR2 (*Bacillus subtilis*) was applied. The lowest value of 7 was observed in the control treatment, where no PGPR or fertilizer amendments were applied. The number of seeds per pod was significantly greater (61.81% and 60.38%) than that in the control treatment because of the combined application of the fully recommended fertilizer (N: P:K@140:55:60) kg/ha with PGPR2 (*Bacillus subtilis*), followed by the application of biochar with PGPR2 (*Bacillus subtilis*), as shown in Table 5.

### Effects on pod length

In year 1, the average pod length of canola was observed to be a maximum of 9.16 cm by the combined application of PGPR2 (*Bacillus subtilis*) with the fully recommended fertilizer, followed by 8.5 cm due to the application of biochar with PGPR2 (*Bacillus subtilis*). The lowest value of 3.66 cm was observed in the control treatment, where no PGPR or fertilizer amendments were applied. The average pod length was 60.04% and 56.94% greater than that in the control treatment because of the combined application of the fully recommended fertilizer (N: P:K@140:55:60) kg/ha with PGPR2 (*Bacillus subtilis*), followed by the application of biochar with PGPR2 (*Bacillus subtilis*), as shown in Table 4. Similarly, in year 2, the average pod length of canola was observed to be a maximum of 9.5 cm by the combined application of PGPR2 (*Bacillus subtilis*) with the fully recommended fertilizer, followed by 9 cm due to the application of biochar with PGPR2 (*Bacillus subtilis*). The lowest value of 4 cm was observed in the control treatment, where no PGPR or fertilizer amendments were applied. The pod length was 57.89% and 55.55% greater than that in the control treatment because of the combined application of the fully recommended fertilizer (N: P:K@140:55:60) kg/ha with PGPR2 (*Bacillus subtilis*), followed by the application of biochar with PGPR2 (*Bacillus subtilis*), as shown in Table 5.

### Effects on biological yield

**Fig. 1:**
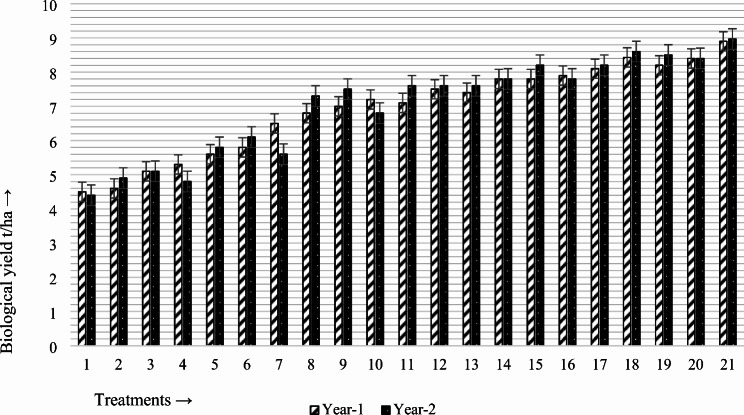
Synergistic effects of plant growth-regulating rhizobacteria (PGPR) and fertilizer amendments on the biological yield (t/ha) of canola

In year 1, the average biological yield of canola was observed to be a maximum of 8.9 t/ha when PGPR2 (*Bacillus subtilis*) was combined with the fully recommended fertilizer, followed by 8.43 t/ha when biochar combined with PGPR2 (*Bacillus subtilis*) was applied. The lowest value of 4.5 t/ha was observed in the control treatment, where no PGPR or fertilizer amendments were applied. The average biological yield was significantly (49.43% and 46.61%) greater than that of the control treatment because of the combined application of the fully recommended fertilizer (N: P:K@140:55:60) kg/ha with PGPR2 (*Bacillus subtilis*), followed by the application of biochar with PGPR2 (*Bacillus subtilis*), as shown in Fig. 1. Similarly, in year 2, the average biological yield of canola was observed to be a maximum of 8.97 t/ha with the combined application of PGPR2 (*Bacillus subtilis*) with the fully recommended fertilizer, followed by 8.6 t/ha with the application of biochar with PGPR2 (*Bacillus subtilis*). The lowest value of 4.4 t/ha was observed in the control treatment, where no PGPR or fertilizer amendments were applied. The biological yield was 50.94% and 48.83% greater than that of the control treatment because of the combined application of the fully recommended fertilizer (N: P:K@140:55:60) kg/ha with PGPR2 (*Bacillus subtilis*), followed by the application of biochar with PGPR2 (*Bacillus subtilis*), as shown in Fig. 1.

### Effects on grain yield

**Fig. 2:**
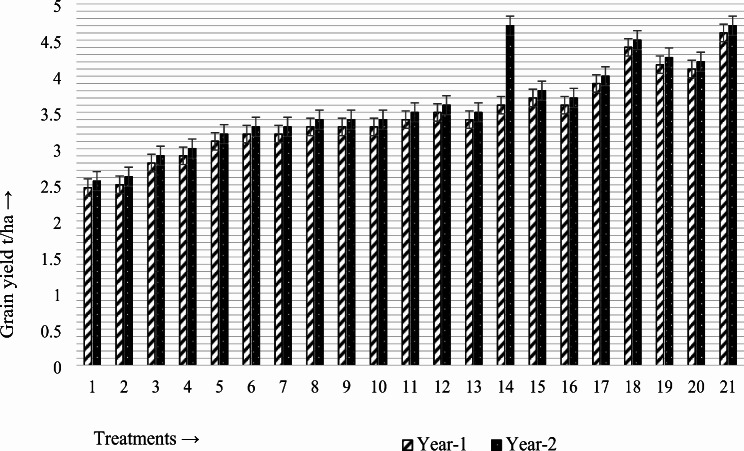
Synergistic effects of plant growth-regulating rhizobacteria (PGPR) and fertilizer amendments on the grain yield (t/ha) of canola

In year 1, the average grain yield of canola was observed to be a maximum of 4.6 t/ha by the combined application of PGPR2 (*Bacillus subtilis*) with the fully recommended fertilizer, followed by 4.4 t/ha due to the application of biochar with PGPR2 (*Bacillus subtilis*). The lowest value of 2.46 t/ha was detected in the control treatment, where no PGPR or fertilizer amendments were applied. The average grain yield was significantly 46.52% and 44.09% greater than that of the control treatment because of the combined application of the fully recommended fertilizer (N: P:K@140:55:60) kg/ha with PGPR2 (*Bacillus subtilis*), followed by the application of biochar with PGPR2 (*Bacillus subtilis*), as shown in Fig. 2.

Similarly, in year 2, the average grain yield of canola was observed to be a maximum of 4.7 t/ha by the combined application of PGPR2 (*Bacillus subtilis*) with the fully recommended fertilizer, followed by 4.5 t/ha due to the application of biochar with PGPR2 (*Bacillus subtilis*). The lowest value of 2.55 t/ha was detected in the control treatment, where no PGPR or fertilizer amendments were applied. The grain yield was 45.74% and 43.33% greater than that of the control treatment because of the combined application of the fully recommended fertilizer (N: P:K@140:55:60) kg/ha with PGPR2 (*Bacillus subtilis*), followed by the application of 2 t/ha biochar with PGPR2 (*Bacillus subtilis*), as shown in Fig. 2.

## Discussion

The use of compost, plant residue mulch, manures, and cover crops increases the content of carbon in the soil and helps improve the soil, which is considered sustainable agriculture and soil conservation [27]. These organic fertilizers help in the sequestration of atmospheric carbon in the soil, increase the fertility of the soil, and further subsidize the ecosystem services of the soil, thereby increasing the strength of the soil and its sustenance [9]. This is indeed the case for plant growth-promoting rhizobacteria (PGPR), which, in the context of sustainable agriculture, support crop productivity and optimize plant nutrients [33]. These PGPRs may act via nitrogen fixation, solubilization of phosphorus, production of indole acetic acid, and other processes via phytohormone production [10]. Thus, PGPR-based inoculants have proven to be potential biotechnological tools for increasing soil fertility and plant productivity, which can be powerful methods for providing food security and rendering agroecosystems sustainable [34]. When used in crops such as legumes, cereals, vegetables, and other crops, PGPRs are vital, particularly under changing climate regimes and the practice of sustainable agriculture [35]. PGPRs are viewed as an influential tool for changing modern agriculture because they provide an opportunity to develop eco-friendly strategies for crop management [36]. Hence, the adoption of PGPR technology makes it possible to develop a healthy food chain for future generations. PGPRs increase the ability of plants to combat diseases and reduce the frequency of watering needed. To some extent, the application of PGPR in agricultural practices can increase the achievement of sustainable development objectives. *Bacillus subtilis* produces and releases bioactive compounds, such as B vitamins, nicotinic acid, pantothenic acid, biotin, and heteroxins, and promotes the growth of plants; gibberellin is needed to stimulate the formation of the root system [37]. Additionally, *Bacillus subtilis* solubilizes inorganic and organic phosphorus, a characteristic of efficient free-living nitrogen-fixing bacteria [38]. Notably, several studies suggest that *Bacillus* species can facilitate plant growth through auxin production, independent of nitrogen fixation [33].

In this study, we observed that the combined application of fully recommended fertilizers (N: P:K @140:55:60) kg/ha with PGPR2 (*Bacillus subtilis*) increased the plant height, number of primary and secondary branches, number of pods, number of seeds per pod, biological yield, and grain yield of canola. This occurred because *Bacillus subtilis*, which enhances the bioavailability of nutrients, reduces the need for synthetic fertilizers, enhances the soil structure, increases water retention, promotes beneficial microbial communities, and produces auxin (indole-3-acetic acid, IAA), a phytohormone essential for plant growth and development [39]. Auxin promotes root elongation; increases root hair and lateral root formation; enhances nutrient uptake; and plays a central role in cell division, elongation, fruit development, and senescence, initiating root, leaf, and flower development [40]. Similarly, Iqbal et al. [4] noted that the coinoculation of plant growth-promoting rhizobacteria with fertilizers increases the growth and yield of canola. Additionally, *Bacillus subtilis* inhibits the growth of plant pathogens and pests; thus, control measures that require the use of chemicals are limited [36]. The present work supports Martínez et al. [41], who concluded that *Bacillus subtilis* improves the physical properties of the soil, increases water availability, and stimulates the growth of healthy microorganisms. Research has also revealed that, through the inoculation of PGPRs, crop yields increase, plant health increases, and soil fertility increases. Furthermore, it inhibits plant pathogens and pests and therefore has chemical control mechanisms [42]. The use of PGPRs has increased the yield, health, and fertility of crops in the areas where they are used [38].

The increase in the height of the plant, primary and secondary branches, number of seeds per pod, pod length, and biological and grain yields of canola were also found to reach a maximum when biochar was applied at a rate of 2 t/ha along with PGPR2 (*Bacillus subtilis*) after treatment (fully recommended fertilizer with *Bacillus subtilis*). This was due to the synergistic effects of PGPR with biochar because biochar application enhances the oxidation‒reduction reactions of the soil matrix, improving soil fertility [1]. Biochar has received much interest as a soil amendment because it is less expensive to produce, and although it is a carbon source, it also improves the chemical properties of the soil. The general use of biochar has been well documented to alter the physical and chemical characteristics of the soil, thus improving plant growth and yield [43]. These benefits are expected, mainly because biochar enhances water and nutrient retention, enhances the cohesiveness and porosity of the soil, enhances nutrient uptake by plants, and stimulates microbial activities in the soil [5]. However, general alterations for specific sort properties and conditions may be positive, although there are always exceptions, and they could even be neutral or slightly negative within certain situations. These findings are supported by Nagah et al. [44], who noted that these increased microorganisms also help counter abiotic stress conditions such as drought and salinity, in addition to eradicating the need for chemical fertilizers and pesticides. *Bacillus* species are also effective at increasing human and soil carbon stocks, decreasing the emission of greenhouse gases, decreasing soil erosion, and increasing the quality of supplied water [14]. The authors of Artyszak & Gozdowski [45] also reported that the application of *Bacillus subtilis* positively influences the growth of plants not only through nitrogen fixation but also through the promotion of root growth, increased mineral absorption and pathogenic fungal and bacterial inhibition.

The amendment of biochar positively affects the stability of the structural components of soil, such as its aggregates, solids, and organic matter, which are favorable for plant growth [46]. The various sizes of particles in the biochar also help increase the water holding capacity and aeration of healthy soil [34]. Additionally, biochar can address poor structure by increasing porosity and soil aeration, especially in compacted ecosystems [47]. Notably, while fine sandy soil has a greater surface area and porosity than biochar does, biochar is a good soil amendment. Biochar mixed with composted biomass also has a positive effect; the quickly decayed biomass provides a stable nitrogen source to plants until nutrients are slowly released from the biochar. Additionally, biochar has a long residence time in the environment and soil and is thus a long-term solution to soil amendments [38]. Lalay et al. [36] reported that in dry agro-environmental settings, biochar (BC) and plant growth-promoting microorganisms (PGPR) may be useful agronomical methods for reducing the effects of drought. Tian et al. [48] reported that in a variety of soil types, biochar has the ability to significantly increase upland crop output and nitrogen usage efficiency (NUE) [49].

## Conclusion and policy implications

Like synthetic fertilizers, organic fertilizers are safer and better for the environment because they are not centralized. Owing to their efficiency in the farming process, they are nonpoisonous and therefore suitable for sustainable farming. In association with the bioeconomy, plant growth-promoting rhizobacteria have numerous positive effects on agriculture. This is crucial for many of the commercially valuable monoculture crops because soil alterations are required for enhanced germination, yields, and disease tolerance. This two-year study assessed the use of PGPRs together with organic and inorganic fertilizers to increase canola productivity and production. Two PGPR strains, *Azotobacter salinestris* and *Bacillus subtilis*, were combined with biochar, compost, animal manure, poultry manure, and NPK fertilizer. The full recommended dose of N: P:K (140:55:40 kg/ha) combined with *Bacillus subtilis* enhanced the production of canola as well as other agronomical characteristics. Compared with the control and other treatments, this combination enhanced most aspects of plant growth and nutrient status; thus, the yields were greater. Furthermore, the present investigation revealed that the interaction of *B. subtilis* and biochar at a rate of 2 tons/ha significantly enhanced canola yield and quality traits. Biochar benefits the microbial quantity and quality of the soil, structure, and nutrient control; this encourages crop-producing capacity and better crop quality. All these strategies provide enduring methods for increasing canola productivity and production. These treatments are capable of increasing the quality of crop production and, consequently, increasing the yields of canola producers, increasing the benefits to producers. On the basis of these findings, we recommend treating canola seeds with *Bacillus subtilis* in combination with either the full recommended dose of N: P:K (140:55:40 kg/ha) or biochar at a rate of 2 t/ha. The above strategies offer positive, long-term approaches to increasing canola production. Canola farmers on the same note may benefit from these treatments since they improve crop performance and, consequently, yield and quality of produce are improved. In conclusion, extending PGPRs via the correct combination of organic and inorganic additions may improve canola production. This study is concerned with the premise that these combinations could highlight ways that may deem farming practices sustainable for growers and the environment. The recommendation to combine chemical fertilizers with PGPRs and organic amendments can align with sustainable agriculture if managed carefully. Using chemical fertilizers at recommended levels alongside organic amendments reduces dependency on chemicals, supports soil health, and enhances nutrient efficiency. This approach can ensure high productivity while minimizing the negative impact on soil microorganisms and reducing environmental harm, thus supporting long-term sustainability. However, excessive reliance on chemical fertilizers may still harm soil biology, so balanced use is crucial.

## Data Availability

The data that support the findings of this study are available on request from the corresponding author M.I.

## Declarations

### Generative AI and AI-assisted technologies in the writing process

During the preparation of this manuscript the author(s) used Chat GPT and Grammarly in order to improve the technical and English language of the paper. After using this tool/service, the author(s) reviewed and edited the content as needed and take(s) full responsibility for the content of the publication.
